# Chimeric antigen receptor (CAR) T therapies for the treatment of hematologic malignancies: clinical perspective and significance

**DOI:** 10.1186/s40425-018-0460-5

**Published:** 2018-12-04

**Authors:** Michael M. Boyiadzis, Madhav V. Dhodapkar, Renier J. Brentjens, James N. Kochenderfer, Sattva S. Neelapu, Marcela V. Maus, David L. Porter, David G. Maloney, Stephan A. Grupp, Crystal L. Mackall, Carl H. June, Michael R. Bishop

**Affiliations:** 1 University of Pittsburgh, Pittsburgh, PA USA; 2 Winship Cancer Institute, Emory University, Atlanta, GA USA; 3 Department of Medicine, Memorial Sloan Kettering Cancer Center, New York, NY USA; 4 Experimental Transplantation and Immunology Branch, National Cancer Institute, Bethesda, MD USA; 5 Department of Lymphoma/Myeloma, The University of Texas MD Anderson Cancer Center, Houston, TX USA; 6 Massachusetts General Hospital/Harvard Medical School, Boston, MA USA; 7 Perelman School of Medicine at the University of Pennsylvania, Philadelphia, PA USA; 8 Clinical Research Division, Fred Hutchinson Cancer Research Center, Seattle, WA USA; 9 Division of Oncology, Cancer Immunotherapy Program, Children’s Hospital of Philadelphia, Philadelphia, PA USA; 10 Cancer Immunology and Immunotherapy Program, Stanford University, Stanford, CA USA; 11 Section of Hematology/Oncology, Department of Medicine, University of Chicago, Chicago, IL USA; 12 The University of Chicago, 5841 S. Maryland Avenue, MC 2115, Chicago, IL 60637 USA

**Keywords:** Chimeric antigen receptor, Tisagenlecleucel, Axicabtagene ciloleucel, Leukemia, Lymphoma

## Abstract

Chimeric Antigen Receptor (CAR) T cell therapies – adoptive T cell therapies that have been genetically engineered for a new antigen-specificity - have displayed significant success in treating patients with hematologic malignancies, leading to three recent US Food and Drug Administration approvals. Based on the promise generated from these successes, the field is rapidly evolving to include new disease indications and CAR designs, while simultaneously reviewing and optimizing toxicity and management protocols. As such, this review provides expert perspective on the significance and clinical considerations of CAR T cell therapies in order to provide timely information to clinicians about this revolutionary new therapeutic class.

## Background

Adoptive cell therapies (ACT) involve collection of immune cells from a patient or a donor, often followed by ex vivo manipulation and/or expansion and reinfusion, have been investigated for decades and are now a cornerstone of cancer immunotherapy. ACTs can be designed to overcome cancer immune evasion mechanisms by directly targeting cancer, thus activating a powerful and (ideally) specific immune response to the tumor. Multiple iterations of ACT are in development for the treatment of cancers, including dendritic cells, natural killer cells, and tumor-infiltrating lymphocytes [1–3]. Initial evidence of responses to ACT using patient-specific tumor infiltrating lymphocytes (TIL) described in the treatment of patients with advanced ovarian cancer, and has since been used to treat patients with melanoma, prostate cancer, and renal cancer, among others [4–6].

Successful ACT depends on adequate numbers of effector cells in the patient, which in turn requires precursors with natural anti-tumor recognition, or engineering of the T cells to provide this recognition. As such, researchers have developed strategies to increase tumor recognition of adoptively stimulated cells outside of T cell receptor (TCR)/major histocompatibility complex (MHC)/peptide recognition. Genetic engineering of novel receptors – now known as Chimeric Antigen Receptors (CAR) – can both recognize cancer-associated antigens and provide T cell activation, proliferation, and memory. CAR constructs are hybrid molecules that can replace major functions of the TCR, including surface antigen recognition as well as T cell activation and costimulation by incorporation of the intracellular domains, most commonly comprising CD3ζ, CD28, and/or 4-1BB [7]. Initial reports of CARs demonstrated successful CAR expression and antigen-specific cytotoxicity in T cells engineered with anti-2,4,6-trinitrophenyl (TNP) CAR constructs [8].

Clinical trials have shown high response rates after anti-CD19 CAR infusion in patients with B cell malignancies, including diffuse large B cell lymphoma (DLBCL) and B cell-precursor acute lymphoblastic leukemia (ALL), resulting in two FDA approved therapies [9, 10]. Studies have also observed specific toxicities – including cytokine release syndrome (CRS) and CAR T cell-related neurotoxicity (NTX) [11]. Based on these data, ongoing pre-clinical and clinical research programs are optimizing CAR T cell strategies in order to increase durability of response, target other cancers, and to control and prevent major toxicities. Our purpose here is to discuss the history of CAR T therapies, review the current toxicity management protocols, and provide an overview of in-development technologies for clinicians who will be treating patients with or referring patients for these groundbreaking therapies.

## Main Text

### CAR design across generations

CARs consist of three major domains: an ectodomain, transmembrane domain, and an endodomain. The ectodomain is the extracellular portion of the receptor that includes the antigen-recognition domain as well as a signal peptide for direction to the endoplasmic reticulum. The transmembrane domain primarily supports CAR stability. The intracellular endodomain facilitates signal transduction to activate T cells during antigen recognition [7].

**Fig. 1:**
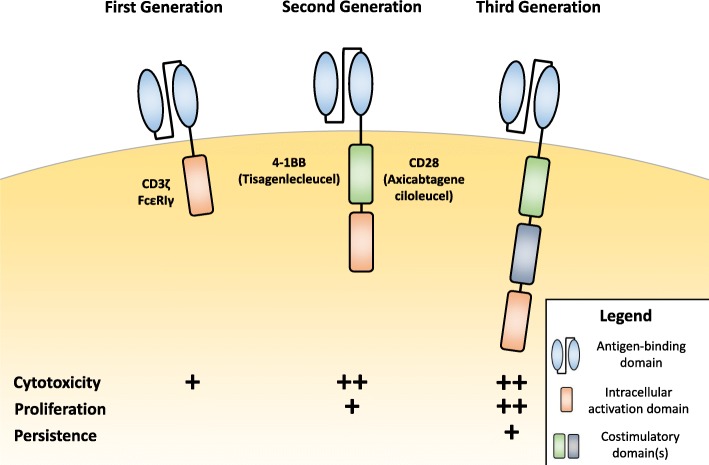
Chimeric Antigen Receptor (CAR) design across generations**.** First generation Chimeric Antigen Receptors (CARs) include an extracellular antigen-binding domain and an intracellular T cell activation domain, commonly CD3ζ or FcεRIγ. Second generation CARs built upon first generation constructs by including an intracellular costimulatory domain, commonly 4-1BB or CD28, incorporated into the FDA approved CAR T therapies tisagenlecleucel and axicabtagene ciloleucel, respectively. Costimulatory domains help enhance CAR T cell cytotoxicity and proliferation compared to first generation designs. Third generation CARs include multiple costimulatory domains, primarily to increase CAR T cell proliferation and persistence

The endogenous TCR complex is expressed on the surface of T cells and can recognize antigens bound to MHC molecules on antigen-presenting cells to activate the T cell [12]. First-generation CARs were designed similarly to the endogenous TCR complex by incorporating a CD3ζ- chain or FcεRIγ intracellular domain, but instead incorporated extracellular antigen-recognition domains that allowed for direct antigen-recognition on the surface of tumor cells, allowing for MHC-independent T cell activation (Fig. 1). Importantly, these first generation designs did not include costimulatory domains to provide the second signal for full T cell activation. First-generation CAR T cells are more susceptible to apoptosis and have limited in vivo expansion and cytotoxicity [7].

In distinction, second-generation CAR constructs incorporate both signal 1 and signal 2 for full T cell activation. Where signal 1 comes from CD3ζ and signal 2 comes from a costimulatory domain to promote interleukin-2 (IL-2) secretion, which promotes T cell activation and prevents apoptosis [7] Multiple costimulatory domains can be utilized to significantly change CAR T cell cytokine secretion profiles to increase cytotoxicity and T cell persistence, as FDA approved CARs incorporate either CD28 or 4-1BB (CD137) [13, 14]. The benefits of a specific costimulatory domain remain under investigation.

Third-generation CAR constructs, which combine multiple intracellular signaling domains, including combinations like CD3ζ-CD28-OX40 and CD3ζ-CD28-41BB are being investigated preclinically [15, 16]. Combination costimulatory domains further support increased cytokine production and could increase efficacy, but there is little clinical data available to assess this.

### CAR T cell production processes

Time from CAR T collection to patient infusion currently takes ~ 3 weeks. Peripheral blood mononuclear cells are collected from the patient using a large-volume leukapheresis procedure. The cells are then transferred to a GMP manufacturing facility for T cell engineering and expansion. Patient T cells are then incubated with CAR-encoding viral vectors, which enter the T cells and introduce CAR gene RNA. CAR RNA is then reverse-transcribed into DNA, which recombines into the T cell genome, resulting in permanent CAR gene incorporation. As this process is not 100% efficient, transformed T cells undergo ex vivo expansion for multiple days, resulting in a product that is ~ 90% CD3+ T cells, but variable fractions of which are CAR+. The cells are transferred back to the center for infusion, which typically happens as a single infusion for FDA approved therapies [13, 14, 17]. CAR T cell infusion recommendations vary across centers, sponsors, and products. Existing protocols include both inpatient and outpatient infusions, and dosage dependent on the total CAR+ population in the infusion product, as well as patient characteristics including age and weight [9, 10]. Of note, recent studies of a responding patient identified a single CAR T cell clone that made up for 94% of the total CAR T cell population [18]. These data suggest that infusion of a single CAR+ T cell can promote therapeutic response.

### Initial CAR T therapy clinical testing

Initial clinical reports on CAR T cell therapeutic efficacy and safety began with a 2010 case report describing a partial response of B cell follicular lymphoma and ablation of blood and bone marrow B cells in a patient treated with a second-generation CD19 CAR T utilizing CD28 [19]. The patient underwent leukapheresis, lymphodepletion with fludarabine and cyclophosphamide (flu/cy), and the patient received the CAR product (infused over 2 days) followed by high-dose IL-2. The patient remained in partial remission for 32 weeks after treatment until discovery of progressive CD19+ lymphoma in lymph nodes. The patient then received chemotherapy plus CAR T cells/IL-2, and remains progression-free at the time of this report (personal communication, [20]). Further early development showed that this anti-CD19 CAR T cell product was highly effective against diffuse large B cell lymphoma, and this product went on to become the FDA approved therapy axicabtagene ciloleucel [9, 13, 21–23].

A report in 2011 described using a second generation, 4-1BB CD19 CAR for chronic lymphocytic leukemia (CLL), demonstrating complete responses (CR) [24]. This product became the first FDA approved CAR T cell therapy, tisagenlecleucel [10, 14]. Three patients with advanced, chemotherapy-resistant CLL (two p53-deficient) received autologous CD19 CAR T cells, with no exogenously administered cytokines. Two CRs (11+ mos and 10+ mos) and one partial response (7 mos) were observed. Toxicities occurred between days 7 and 21 in all patients, but all cases were reversible and treatable. Importantly, researchers noted that one patient had measurable CAR-expressing T cells up to 6 months post-treatment, and in vitro characterization confirmed that CAR T cell effector function was retained [24, 25]. Follow-up of these two patients show them to be in continued CR for > 5 years, with continued B cell aplasia suggesting continued functional persistence of the CAR T cells (personal communication).

Following pre-clinical studies showing efficacy of CD19 CAR T cells against ALL, the first data showing efficacy in relapsed/refractory ALL patients were published, with CRs observed responses in two children and five adults in separate studies, including patients with chemorefractory disease. Of the 5 adult patients who completed treatment, all were minimal residual disease (MRD)-negative and four underwent subsequent allogenic stem cell transplant (SCT). One patient died in CR post allo-BMT due to a pulmonary embolus [26]. The pediatric report demonstrated the role of IL-6 blockade in controlling CRS, and also showed that a mechanism of relapse is CD19 escape with CR functional persistence [27]. Pediatric patient 1 experienced a durable CR that corresponded with CAR T cell expansion, had severe CRS relieved by the IL-6 receptor blocking agent tocilizumab, and remains in remission in B cell aplasia without SCT 6 years later (personal communication). Unfortunately patient 2, who had a CR one month after CD19 CAR infusion after failure to respond to blinatumomab, relapsed after two months with CD19- ALL [27].

### Current FDA CAR T approvals

To date, two CAR T therapies have been granted three total FDA approvals for the treatment of patients with hematologic malignancies. The first approval - granted on August 30th, 2017 – was awarded to 4-1BB-based CD19 CAR T cell therapy tisagenlecleucel (CTL019, Kymriah, Novartis, Basel, Switzerland) for the treatment of patients up to 25 years of age with ALL that is refractory or in second or later relapse [10]. Patients enrolled in the phase II global ELIANA registration trial (NCT02435849) were between the ages of three and 23 (*n* = 75) and had not received prior anti-CD19 therapy. All patients who were not leukopenic (72/75 patients) received lymphodepletion regimen before receiving a median dose of 3.1 × 10^6^ cells/kg CAR T cells. Data supporting FDA approval is listed in Table 1. At publication of trial results, the overall remission rate in patients who received infusions of CAR T cells was 81% (95% CI: 71–89), and the median duration of remission had not yet been reached [17].

**Table 1:** Supporting data for FDA approval of tisagenlecleucel for r/r B-ALL (≤ 25 years)

Drug	Tisagenlecleucel
Indication	r/r B-ALL (≤ 25 years)
Clinical Trial	ELIANA (NCT02228096)
Overall Survival (N)	75
12-month OS (%)	76
95% CI	(63–86)
Median OS (mos)	19.1
95% CI	(15.2 – NE)
Event-free Survival (N)	73
12-month EFS (%)	50
95% CI	(35–64)
Duration of Remission (N)	61
Median (mos)	NR

*Adapted from 10, 17. Abbreviations: *OS* Overall survival, *CI* Confidence interval, *Mos* Months, *EFS* Event-free survival

On October 18th, 2017, the FDA granted a second CAR T therapy approval to the CD28-based CD19 CAR T cell product axicabtagene ciloleucel (Axi-cel, Yescarta, Kite Pharma/Gilead, Los Angeles, CA) for the treatment of patients with DLBCL who have not responded or have relapsed after two prior treatment regimens [9]. 101 patients (77 DLBCL, 24 other) in the phase I/II ZUMA-1 trial (NCT02348216) received autologous CAR T cells (2 × 10^6^ cells/kg) after flu/cy lymphodepletion. Data supporting FDA approval is listed in Table 2. At the time of publication, an objective response rate (ORR) of 82% (95% CI: 73–89) was observed, median time to response was 1 month (95% CI: 0.8–6), and the median duration of response was 8.1 months (95% CI: 3.3 – no estimate). 18-month overall survival (OS) was 52% [13].

**Table 2:** Supporting data for FDA approvals of axicabtagene ciloleucel and tisagenlecleucel for r/r DLBCL

Drug	Axicabtagene ciloleucel	Tisagenlecleucel
Indication	r/r DLBCL (adult)	r/r DLBCL (adult)
Clinical Trial	ZUMA-1 (NCT02348216)	JULIET (NCT02445248)
Patients Treated (N)	101	68
Objective Response Rate (N, %)	73 (72%)	34 (50%)
95% CI	(62–81)	(37.6–62.4)
Complete Response Rate (N, %)	52 (51%)	22 (32%)
95% CI	(41–62)	(21.5–44.8)
Partial Response Rate (N, %)	21 (21%)	12 (18%)
95% CI	(13–30)	(9.5–28.8)
Median Duration of Response (mos)	9.2	NR
95% CI	(5.4 – NR)	(5.1 – NR)
Median Follow-up (mos)	7.9	9.4
Median Duration of Response for CR (mos)	NR	NR
95% CI	(8.1 – NR)	(10.0 – NR)
Median Duration of Response for PR (mos)	2.1	3.4
95% CI	(1.3–5.3)	(1.0 – NR)

*Adapted from 9, 10, 13, 14. Abbreviations: *CI* Confidence interval, *Mos* Months, *CR* Complete response, *PR* Partial response

On May 1st, 2018, tisagenlecleucel gained a second FDA approval, this time for the treatment of adult patients with relapsed/refractory DLBCL [10]. This approval was based on results from the phase II JULIET clinical trial (NCT02445248) where 81 evaluable patients (age 22–76 years, median = 56 years) were treated with tisagenlecleucel (1.0 × 10^7^–6.0 × 10^8^ CAR T cells) after lymphodepletion chemotherapy or prior leukopenia. Data supporting FDA approval is listed in Table 2. At time of publication, patient population ORR was 53.1% (CR = 39.5%, PR = 13.6, 95% CI: 42–64, *p* < 0.0001), and 6-month OS was 64.5% (95% CI: 51.5–74.8). Anti-CD19 CAR T cells were detected in responder blood for up to 367 days post-infusion. In 99 evaluable patients, CRS incidence was 58% (23% grade 3–4), and NTX incidence was 21% (12% grade 3–4). No deaths were reported due to therapy [14].

### Lymphodepletion regimens prior to CAR T therapy

Studies indicate that patient lymphodepletion prior to CAR T therapy can improve clinical outcomes by increasing CAR T cell expansion and persistence. Tisagenlecleucel and axi-cel clinical trials included lymphodepletion regimens prior to patient CAR T infusion [13, 14, 17]. By far the most commonly used lymphodepletion regimens include flu/cy, although at a variety of doses. The cyclophosphamide dose commonly used ranges from 900 mg/m^2^ to 3600/m^2^ total dose. While the ELIANA trial testing tisagenlecleucel efficacy in patients with ALL used flu/cy in almost all patients, the JULIET trial of the same product in DLBCL utilized individualized lymphodepletion strategies based on patient therapeutic response history, blood cell counts, and organ function [14, 17]. Patient response to tisagenlecleucel was consistent across the overall patient population. Concerning axi-cel, patients enrolled in the ZUMA-1 trial received a fixed dose of fludarabine (30 mg/m^2^) and cyclophosphamide (500 mg/m^2^) on days 5, 4, and 3 pre-infusion [13]. Of note, some trials forgo lymphodepletion in patients who are already leukopenic with no evidence of detriment.

### CAR T therapy toxicities

Toxicities observed in patients who receive CAR T therapies usually present within days of first infusion. The two most common toxicities are CRS and NTX, either of which can be lethal [28].

#### Cytokine release syndrome (CRS)

##### Overview

CRS is a condition hallmarked by increased cytokine production and associated inflammation. CRS generally correlates with increased IFNƴ,GM-CSF, IL-10, and IL-6, as well as CAR T cell expansion [29]. CRS clinical presentation includes fever, nausea, anorexia, tachycardia and/or hypotension, cardiac dysfunction, renal impairment, and hepatic failure among other conditions [29, 30]. Higher disease burdens correlate with greater CRS severity, especially in ALL [11]. Anti-tumor activity of CAR T therapies, however, is not predicated by disease severity, as patients who do not experience CRS may still respond to therapy [11].

##### CRS grading

Multiple grading scales for CAR T-related CRS have been developed. Among the most widely used is a system that reflects the potential severity of CRS in some CAR T cell patients (Fig. X) [29]. Using this scale, grade 1 CRS symptoms include non-life threatening events including fever and nausea. Grade 2 symptoms include oxygen requirement < 40%, hypotension that responds to fluids or low dose vasopressor, and grade 2 organ toxicity. Grade 3 CRS symptoms include an oxygen requirement ≥40%, hypotension requiring high-dose or multiple vasopressors, grade 3 organ toxicity, and/or grade 4 transaminitis. Grade 4 CRS include life-threatening symptoms that require ventilator support or grade 4 organ toxicity, excluding transaminitis [29].

##### CRS treatment

CRS treatment algorithms may utilize CRS grading scales or clinical factors, and work is underway to try to harmonize both CRS grading and treatment approaches. Grade 1 CRS often requires supportive care. Treatment of grade 1 CRS with corticosteroids is not recommended due to potential inhibitory activity upon CAR T cells. Nonsteroidal anti-inflammatory drugs for fever are also not recommended due to potential risk of hemorrhage in thrombocytopenic and/or coagulopathic patients [29]. Some patients with Grade 2 CRS and essentially all patients with Grade 3/4 CRS are treated with the IL-6 receptor agonist tocilizumab. Clinical trial results from the University of Pennsylvania/ Children’s Hospital of Philadelphia, as well as the ELIANA trial indicate that CRS severity specifically correlated with patient IL-6 levels [17]. As a result, the FDA concurrently approved tocilizumab with tisagenlecleucel for the treatment of CAR T-associated CRS. Subsequent studies have confirmed tocilizumab efficacy in reversing CRS symptoms, as well as having little to no inhibitory effect upon CAR T therapeutic efficacy [28, 31]. A stepwise CRS treatment algorithm uses tocilizumab first line. If severe CRS continues (especially hypotension), a short course of corticosteroids can be used [30]. Axi-cel specifically recommends 1 mg/kg methylprednisolone or equivalent dexamethasone for grade 3 CRS, and 1000 mg methylprednisolone at grade 4 [9].

#### Neurotoxicity (NTX)

##### Overview

NTX, which can present as CAR T-related encephalopathy (CRES), includes a variety of neurological symptoms - including confusion, delirium, expressive aphasia, obtundation, myoclonus, and seizure. White matter degradation has also been observed in some severe cases [11, 29, 30, 32]. To date, causality of CRES is unknown, but studies suggest that cytokine secretion and subsequent breakdown of the blood-brain barrier may play a role [33]. Of note, patients treated with blinatumomab – a bi-specific anti-CD19/CD3 BiTE antibody – also have increased incidence of neurological toxicities similar to those observed in patients treated with CAR T [34].

##### Grading

The University of Texas MD Anderson Cancer Center has developed a grading scale for CRES [32]. All CRES grades include a score from the CARTOX 10-point neurological assessment, where 1 point is assigned for successful completion of a basic neurological function (Fig. X). Grade 1 CRES is classified as a patient with CARTOX scores 7–9. Grade 2 CRES includes patients with CARTOX scores 3–6. Grade 3 CRES includes patients with CARTOX scores 0–2, stage 1/2 papilledema with < 20 mmHg CSF opening pressure, and/or partial seizure or non-convulsive seizures that respond to benzodiazepine. Grade 4 CRES is considered critical with a CARTOX score of 0, stage 3/4/5 papilledema, > 20 mmHg CSF opening pressure, and generalized seizures, convulsive or non-convulsive epilepticus, or motor weakness after CAR T infusion [32].

##### CRES treatment

The above grading scale also has corresponding treatment recommendations (Fig. X) [32]. Grade 1 CRES treatment includes supportive care and consistent neurological evaluation. Grade 2 CRES also requires a neurological evaluation, as well as tocilizumab administration if symptoms are associated with CRS. Unlike CRS where tocilizumab has a clear role, the efficacy of tocilizumab in reversing CRES is not well established. Grade 3/4 CRES requires ICU transfer, and some centers utilize tocilizumab administration if associated with CRS. Corticosteroids - including dexamethasone or methylprednisolone - are also recommended for grade 3/4 CRES [32].

### Other CAR T-associated adverse events

#### B cell aplasia

CD19 CAR T therapies can result in short or long-term patient B cell aplasia, which is also a marker of functional persistence of the CAR T cells [28]. Short-term B cell aplasia may not require treatment, while longer term B cell aplasia may require immunoglobulin replacement, especially in children.

#### *Graft* vs *host disease* (GVHD)

Patients who have had prior stem-cell transplant often retain full donor chimerism in the T cell compartment, so the “autologous” T cells obtained from the patient are usually of donor origin. CAR T clinical trials that supported FDA approvals did not enroll patients with active GVHD requiring systemic therapy [13, 14, 17]. In the absence of active GVHD, the donor origin cells obtained from the patient appear to be tolerated, and GVHD is either not seen or extremely rare.

#### Off-target toxicities

CAR constructs are designed to recognize specific markers, and healthy tissue presenting selected targets may be affected. Targets are optimized through pre-clinical research and early phase clinical trials.

### Other clinical considerations for CAR T therapies

#### Treatment scheduling around stem cell transplant

Many studies have reported patients receiving hematopoietic stem cell transplant (HSCT) post-CAR T therapy to prevent relapse. Across these studies, durability of response between patients who went on to receive allo-HSCT vs those who did not are not statistically significant. These data indicate that CAR T therapy may be beneficial regardless of allo-HSCT consolidation [28, 31, 35, 36]. Whether a patient has MRD- status, however, may suggest a benefit for receiving allo-HSCT after CAR T therapy. One study assessed risk-of relapse post HSCT in patients with relapsed/refractory B-ALL who achieved MRD- after either anti-CD19 or anti-CD22 CAR T therapy. 24-month cumulative incidence of post-HSCT relapse of 25 evaluable patients was 13.5% (95% CI: 3.2–32.1), while 80% of patients who did not receive HSCT (*n* = 20) relapsed post-CAR T infusion [37]. Considering safety, incidence of graft vs host disease in patients who had prior CAR T therapy was consistent with historical rates of HSCT alone [37]. Future studies will need to determine whether CAR T therapy enhances response to HSCT, and whether specific CAR T therapies may be more likely to require HSCT post-infusion to prevent relapse.

#### Patient access to CAR T therapies

CAR T therapies require a significant logistical pipeline incorporating collection of patients leukocytes, engineering of CAR T cells, transportation of both the original and engineered cells, and patient infusion [38]. As of now, only specific quaternary care centers across the world are capable or certified to offer CAR T cell therapies to patients, and even fewer GMP cell manufacturing laboratories are capable of generating CAR T products. Center limitation presents two challenges. Time until CAR T therapy administration could become problematic as new FDA indications are granted or if, disregarding therapeutic cost, clinicians start to use therapies off-label in earlier disease settings.

There are very few treatment options available for relapsed/refractory pediatric ALL. Tisagenlecleucel is currently approved by the FDA for treating patients up to 25 years of age, and thus represents a critical option for this patient population [10]. Immune checkpoint inhibitors, for example, are usually not an option for pediatric patients, as they often require PD-L1 positivity or increased tumor mutational burden for on-label administration and efficacy. Checkpoint blockade, thus far, has shown limited efficacy in treating pediatric cancers [39].

While not fully within the scope of this manuscript, it is worth mentioning that price of CAR T therapies could become limiting, especially if clinicians start to recommend off-label treatments in earlier disease settings. One mechanism in place to relieve cost concerns is attached to tisagenlecleucel, in which the parent company Novartis will not charge patients who did not have evidence of response to treatment within one month post-infusion [40]. Because of rapid advancements in this field, it will also be imperative for clinicians to standardize treatment scheduling of CAR T therapies and inform insurance companies of new standard-of-care therapies to avoid incorrect patient charges.

### Future advances for CAR T therapies

#### Improvements upon FDA approved CAR T strategies

Despite the recent success of CAR T therapies in the clinic, FDA approved therapies can be improved upon concerning increased efficacy and CAR T proliferation/persistence, lowering the potential for acquired resistance, and reducing the risk for severe toxicities. Current therapeutic strategies have demonstrated that CD19 is a viable target for elimination of various hematologic malignancies, but improvements concerning T cell phenotypic abundance, use of alternative costimulatory domains, and combining anti-CD19 CAR T cells with other therapies are currently being tested in numerous ongoing clinical trials. Among the most advanced CAR T therapy currently in development is lisocabtagene maraleucel (liso-cel, Juno Therapeutics/Celgene, Seattle, WA) – formerly known as JCAR017 – that is being evaluated for safety and efficacy in the phase 1 TRANSCEND study (NCT02631044) for the treatment of patients with relapsed/refractory DLBCL. Liso-cel utilizes an anti-CD19 second generation CAR construct with a 4-1BB costimulatory domain. In December 2017, 91 total patients (ECOG PS 0–2, no previous autologous stem cell transplant) were treated with liso-cel. The ‘core’ group of patients – patients with DLBCL and ECOG PS 0–1 – were separated into three dosage cohorts. Patients within the DL2 cohort were treated with 100 million cells (*n* = 29), patients within the DL1 cohort were treated with 50 million cells (*n* = 34), and 4 patients were treated with 50 million cells twice, with 2 weeks between infusions (*n* = 4). Across doses within the core group, 6 month ORR was 92%, with 80% of patients with CR at 3 months remaining in remission at 6 months. Across the trial population, CRS was observed in 35% of patients (1% grade 3–4) and 19% experienced NTX (12% grade 3–4) [41].

### New disease settings for CAR T therapies

Multiple ongoing clinical trials are evaluating CAR T therapies in disease settings beyond B-ALL and DLBCL. Among the most advanced data to date concern CAR T therapies for the treatment of patients with multiple myeloma and chronic lymphocytic leukemia.

#### Multiple myeloma

B cell maturation antigen (BCMA) has been identified as a CAR target for the treatment of multiple myeloma and were first tested in a single-center clinical trial [42–44]. A different anti-BCMA CAR T-cell product known as bb2121 (Bluebird Bio/Celgene) has progressed to a multicenter clinical trial, and on November 16th 2017, the FDA granted a breakthrough designation to bb2121 for the treatment of patients with relapsed/refractory multiple myeloma [45]. Bb2121 is a second-generation CAR construct that contains an anti-BCMA antigen-recognition domain and an intracellular 4-1BB costimulatory domain [45]. BCMA – a member of the tumor necrosis factor receptor superfamily – is required for long-term survival of plasma cells and is often present on multiple myeloma cells [46]. Preliminary results from the phase I CRB-401 study (NCT02658929) involving bb2121 were presented in June 2017. In all, 21 patients (3 prior treatments or double refractory, ≥50% BCMA expression) underwent leukapheresis and subsequent cyclophosphamide/fludaribine lymphodepletion, and then received between 5 × 10^7^ and 1.2 × 10^9^ anti-BCMA CAR T cells. ORR across all patients and doses was 89% (75% PR, 27% CR, 95% CI: 65–99). All patients with CR were MRD-. DOR was > 134 days (95% CI: 7–361). Anti-BCMA CAR T cell expansion was observed, and CAR T cells were measurable in patient blood for up to 24 weeks post-infusion. 15 total cases of CRS were observed, with two grade 3 cases. No grade 3/4 NTX were observed [47]. An update in December 2017 reported an ORR of 94% and a 56% CR rate. Additionally, median PFS was not yet reached after 40 weeks of follow up. The phase 2 KarMMa trial (NCT03361748) is ongoing and will serve as the basis for regulatory submission to the FDA [48].

Similar to bb2121, LCAR-B38M – an anti-BCMA CAR T therapy (Legend/GenScript Biotech, Nanjing, China) – has been evaluated for efficacy in patients with multiple myeloma. Data presented in June 2017, 33/35 patients achieved CR, with 14/19 evaluable patients with at least four months of follow up remaining in remission [49]. LCAR-B38M is currently in phase 1/2 clinical trials (NCT03090689). Additionally, data concerning LCAR-B38M has fueled the development of the anti-BCMA CAR T therapy JNJ-68284528 (Janssen/Johnson & Johnson, Beerse, Belgium) that is currently being evaluated in a phase 1b/2 clinical trial (NCT03548207) for the treatment of patients with relapsed/refractory multiple myeloma [50].

#### Chronic lymphocytic leukemia

Among the first studies investigating anti-CD19 CAR T therapeutic efficacy was a case report treating a patient with CLL. Studies involving tisagenlecleucel for the treatment of patients with CLL reveal a 57% ORR (8/14) with 4 CR. Responses have been durable, with patients with CR remaining disease-free 40 months post-infusion [51]. Since this initial trial, over 60 patients with relapsed/refractory CLL have been treated with anti-CD19 CAR T cells at the University of Pennsylvania and response continues to be significant (personal communication). Interestingly, clinical trial data suggests that combination ibrutinib - a small molecule targeting Bruton’s tyrosine kinase on B cells – may improve tisagenlecleucel efficacy in treating patients with relapsed/refractor CLL. CR was observed in 11 patients at follow up (3–12 months) who received combination therapy. Additionally, no detectable CLL was observed in 10/11 patients at 3 months [52].

#### Acute myeloid leukemia

Phase 1 clinical trials assessing efficacy of various CAR T strategies in patients with acute myeloid leukemia (AML) are also underway. As CD19+ AML is considered rare, alternative antigens – including CD33, CD38, CD56, CD117, CD123, Lewis-Y, Muc-1, and NKGDL – are being considered as targets for developmental CAR T strategies [53–56]. Patient response has been observed in early results. In one example, six adult patients with relapsed AML were treated with anti-CD123 CAR T cells at two doses: 50 × 10^6^ or 100 × 10^6^ cells. In all, one patient receiving 50 × 10^6^ cells experienced MRD-level disease response, two patients receiving 100 × 10^6^ cells achieved CR, and two patients receiving 100 × 10^6^ cells achieved PR [57].

#### Solid tumors

CAR T therapies offer significant promise in treating a variety of solid tumors, but many challenges concerning optimal cellular targets, tumor immune resistance, and toxicities will need to be resolved. We recognize the potential of this ongoing research, but further discussion of these treatments is beyond the scope of this manuscript.

### Expanded indications for FDA approved anti-CD19 CAR T therapies

Axi-cel is also being tested in alternative disease settings. The most advanced data is from the phase 1/2 ZUMA-3 trial (NCT02614066) testing efficacy and safety in the treatment of adult patients with relapsed/refractory ALL (*n* = 23). At median follow-up (2.7 months), CR rate was at 71%, and 17 patients were MRD-. Grade 3/4 CRS was observed in 22% of patients, and grade 3/4 NTX occurred in 12% of patients. Two deaths were reported due disease progression. This trial is ongoing [58].

### New CAR designs

Next-generation CAR T constructs are being designed to help overcome a variety of potential issues including elimination of pre-infusion leukodepletion, overcoming inhibitory factors within the tumor microenvironment, and reducing the risk of relapse. Additionally, CAR T cells constructs are being developed to target antigens other than CD19 on hematologic malignancies.

#### Targeting alternative antigens

CAR constructs targeting antigens other than CD19 are currently being developed. Among the most heavily studied alternative targets for the treatment of hematologic malignancies are CD20, CD22, and CD30. Anti-CD20 CAR T therapies in a phase 1/2a clinical trial (NCT01735604) have thus far demonstrated similar response rates (ORR = 81.8%) in patients with non-Hodgkin lymphoma (*n* = 11) compared to anti-CD19 CAR T therapies after 5 years of follow up [59]. Phase I clinical trial results assessing efficacy of anti-CD22 CAR T cells in treating patients with B-ALL who are naïve or resistant to anti-CD19 CAR T therapy reported a 73% complete remission rate in evaluable patients (11/15) [60]. Anti-CD30 CAR T therapies have also induced complete responses in patients with Hodgkin lymphoma (2/7) or anaplastic large cell lymphoma (1/2) in phase I clinical trials [61].

#### Bi-specific CAR T cells

While anti-CD19 CAR T therapies provide initial complete responses, a significant number of patients relapse, partially due to loss of the targeted antigen. As such, it has been hypothesized that CAR constructs that target more than one antigen may reduce the risk of relapse. Based on studies involving single targeted antigens, clinical trials are underway assessing efficacy of anti-CD19/CD22 (NCT03448393) and anti-CD19/CD20 bi-specific CAR T cells (NCT03271515) to overcome relapse due antigen loss, and to provide treatment options to patients with refractory disease to antigen-specific targeted therapies.

#### IL-12 secreting CAR T cells

Building upon second-generation CAR T constructs, it has been hypothesized that additional IL-12 – a cytokine normally generated from antigen-presenting cells that promotes CD8+ T cell activation through increased IFNƴ secretion – may further improve CAR T cell proliferation and anti-tumor activity, as well as assist in overcoming inhibitory factors associated with the tumor microenvironment [62]. Systemic IL-12 immunotherapies showed promising antitumor activity in pre-clinical models as a monotherapy and in combination with other agents. Clinical trials assessing antitumor efficacy of systemic IL-12, however, failed due to severe toxicities including hematologic and hepatic dysfunction [63–65]. As such, researchers constructed IL-12 secreting anti-CD19 CAR T cells – termed ‘armored’ CAR T cells – and showed enhanced proliferation and increased cytotoxicity compared to non-IL-12 secreting variants when exposed to cell-free ascites from human ovarian tumor-bearing mice [66].

Both pre-clinical and clinical data suggest that leukodepletion is important for effective CAR T cell antitumor activity. Studies are investigating whether the addition of an IL-12 secretion domain in CAR constructs may also eliminate the requirement for leukodepletion. In support, IL-12 secreting CAR T cells were able to clear lymphoma from lymphoreplete mice, while non-IL-12 secreting CAR T cells were unable to do so [67].

#### Targeting T cell metabolism

Towards the goal of improving efficacy and reducing toxicities of cancer immunotherapies, many research programs are working to better understand T cell metabolism and the impact it may have on differentiation, effector function, and interactions with cancerous cells in the tumor microenvironment [68, 69]. For example, one report notes that in vitro 4-1BB costimulation enhances T cell mitochondrial activity that promotes anti-PD-1 response [70]. As CAR T cells retain innate metabolic programming compared to their non-CAR variants, manipulation of CAR T cell metabolism is a potentially viable mechanism to increase effector function and promote proliferation/persistence. While studies are ongoing, preliminary in vitro experiments suggest that CAR T cells with a 4-1BB costimulatory domain generally rely upon oxidative metabolism and display enhanced persistence compared to CD28 CAR T cells that display glycolytic metabolic function [71].

### Next-generation CAR T engineering

#### Using CRISPR in CAR T cell generation

Current CAR T cell engineering methods do not control for CAR gene localization within the T cell genome. Recent developments in Clustered Regularly Interspaced Short Palindromic Repeats (CRISPR) genetic techniques – derived from a bacterial anti-viral defense system – now allow for precise integration of genetic material into target cell genomes [72]. As such, studies are currently underway assessing efficacy of CRISPR-generated CAR T cells. One study notes that CRISPR/Cas9-driven CAR localization to the T cell receptor α constant (TRAC) genomic location allows for uniform CAR expression across all generated T cells, as well as increased cytotoxicity in a murine acute lymphoblastic leukemia model [73].

#### Safety switches

Multiple ‘safety switches’ are being tested in CAR constructs in order to reduce toxicities through more precise control of cell proliferation and activity. For example, one concept being tested incorporates caspase-9/human FK506-binding protein-hybrids that may promote CAR T cell apoptosis in the presence of a synthetic molecule that can be administered to patients after tumor eradication [74]. Other techniques include antibody-based control of CAR T cell activity, as well as the engineering of regulatory mechanisms to control CAR T cell gene expression via a secondary medication [75, 76].

#### Controlling CAR T product composition

Biomarkers are becoming increasingly important for evaluating patient prognosis and predicting immunotherapy success. As CAR T cells present biomarkers of their own, characterization of infusion product functionality and immune marker composition may help identify specific cellular mixes that allow for both anti-cancer activity as well as reduced toxicities. Liso-cel pre-infusion products, for example, are analyzed and subsequently balanced for CD4+ and CD8+ cells prior to CAR transfection [41].

#### Allogeneic ‘off the shelf’ CAR T cells

The development of universal, allogenic CAR T cells – cells engineered from a single donor source that can be used in multiple patients – could help expand CAR T cell therapy access, reduce time to first infusion, and provide product composition consistency towards reducing toxicity and increasing efficacy [77]. Multiple hurdles remain concerning this technology, most notably the ability to overcome potential graft vs host disease and rejection. Allogenic CAR T cells are currently being tested in phase I clinical trials.

## Conclusion

CAR T cell therapies have offered significant promise in the treatment of patients with hematologic malignancies, providing a foundation for the development of treatment strategies for other cancers. FDA approvals for CAR T therapeutic development has moved from bench to bedside more quickly compared to other immunotherapies. While it is important to be able to offer these life-changing therapies to patients as soon as possible, many questions have been left unanswered for the field to discern along the way. Additionally, as toxicities are better understood and treatment scheduling and dosage is further optimized with clinical data, technologies will be continually refined and improved upon. It is clear that CAR T cell therapies are heavily entrenched in the future of cancer immunotherapy. With the unprecedented speed of this field, however, researchers and clinicians must remain vigilant in providing information to clinicians about the multiple caveats of this therapeutic class, allowing for the best patient outcomes possible.

## Abbreviations

ACT: Adoptive cell therapy
ALL: Acute lymphoblastic leukemia
BCMA: B cell maturation antigen
CAR: Chimeric antigen receptor
CI: Confidence interval
CLL: Chronic lymphocytic leukemia
CR: Complete response
CRES: *CAR T* cell-related encephalopathy syndrome
CRISPR: Clustered Regularly Interspaced Short Palindromic Repeats
CRS: Cytokine release syndrome
DLBCL: Diffuse large B cell lymphoma
FDA: U.S. Food and Drug Administration
Flu/cy: Fludarabine/cyclophosphamide
GM-CSF: Granulocyte macrophage colony stimulating factor
GMP: Good manufacturing practice
GVHD: Graft vs host disease
HSCT: Hematopoietic stem cell transplant
IFN: Interferon
IL: Interleukin
MHC: Major histocompatibility complex
MRD: Minimal residual disease
NTX: Neurotoxicity
ORR: Objective response rate
OS: Overall survival
PR: Partial response
SCT: Stem cell transplant
TCR: T cell receptor
TIL: Tumor infiltrating lymphocytes
TNP: Trinitrophenyl
